# Optimization of Intrinsic ZnO Thickness in Cu(In,Ga)Se_2_-Based Thin Film Solar Cells

**DOI:** 10.3390/ma12091365

**Published:** 2019-04-26

**Authors:** Salh Alhammadi, Hyeonwook Park, Woo Kyoung Kim

**Affiliations:** School of Chemical Engineering, Yeungnam University, 280 Daehak-ro, Gyeongsan 38541, Korea; salehalhammadi.1987@gmail.com (S.A.); greatekal@naver.com (H.P.)

**Keywords:** Cu(In,Ga)Se_2_, solar cell material, CIGS, intrinsic ZnO, i-ZnO, shunt resistance

## Abstract

The typical structure of high efficiency Cu(InGa)Se_2_ (CIGS)-based thin film solar cells is substrate/Mo/CIGS/CdS/i-ZnO/ZnO:Al(AZO) where the sun light comes through the transparent conducting oxide (i.e., i-ZnO/AZO) side. In this study, the thickness of an intrinsic zinc oxide (i-ZnO) layer was optimized by considering the surface roughness of CIGS light absorbers. The i-ZnO layers with different thicknesses from 30 to 170 nm were deposited via sputtering. The optical properties, microstructures, and morphologies of the i-ZnO thin films with different thicknesses were characterized, and their effects on the CIGS solar cell device properties were explored. Two types of CIGS absorbers prepared by three-stage co-evaporation and two-step sulfurization after the selenization (SAS) processes showed a difference in the preferred crystal orientation, morphology, and surface roughness. During the subsequent post-processing for the fabrication of the glass/Mo/CIGS/CdS/i-ZnO/AZO device, the change in the i-ZnO thickness influenced the performance of the CIGS devices. For the three-stage co-evaporated CIGS cell, the increase in the thickness of the i-ZnO layer from 30 to 90 nm improved the shunt resistance (R_SH_), open circuit voltage, and fill factor (FF), as well as the conversion efficiency (10.1% to 11.8%). A further increas of the i-ZnO thickness to 170 nm, deteriorated the device performance parameters, which suggests that 90 nm is close to the optimum thickness of i-ZnO. Conversely, the device with a two-step SAS processed CIGS absorber showed smaller values of the overall R_SH_ (130–371 Ω cm^2^) than that of the device with a three-stage co-evaporated CIGS absorber (530–1127 Ω cm^2^) ranging from 30 nm to 170 nm of i-ZnO thickness. Therefore, the value of the shunt resistance was monotonically increased with the i-ZnO thickness ranging from 30 to 170 nm, which improved the FF and conversion efficiency (6.96% to 8.87%).

## 1. Introduction

Among the renewable and sustainable energy sources, solar electricity has attracted considerable attention, and significant progress has been achieved over the last few decades. In addition to conventional wafer-based crystalline Si solar cells and modules which are dominant in the market, compound semiconductor-based thin film solar cells, such as CdTe [1] and Cu(In,Ga)Se_2_ (CIGS) [2], have been successfully commercialized. In particular, the chalcopyrite CIGS thin-film solar cells have demonstrated excellent properties among photovoltaic technologies, such as a high electric conversion efficiency (23.35%) [3], roll-to-roll flexibility [4,5], and excellent outdoor performance stability because the CIGS thin-film solar cells are deemed the most promising photovoltaic technology for future energy [6,7].

Even though diverse fabrication processes of CIGS light absorbers have been successful with efficiencies close to 20%, the most successful processes with greater than 20% electrical conversion efficiency are three-stage co-evaporation (22.6% by Zentrum für Sonnenenergie- und Wasserstoff-Forschung Baden-Württemberg (ZSW)) [8] and two-step metallization-selenization processes (23.35% by Solar Frontier) [3]. The microstructural characteristics of the CIGS absorber, including the density, grain boundaries, grain size, and surface smoothness/roughness, are dependent on the fabrication processes [9]. For example, the CIGS absorber prepared by a three-stage co-evaporation process exhibited a smooth and dense surface morphology, while the CIGS obtained from a two-step metallization-selenization process exhibited high surface roughness [10].

**Figure 1:**
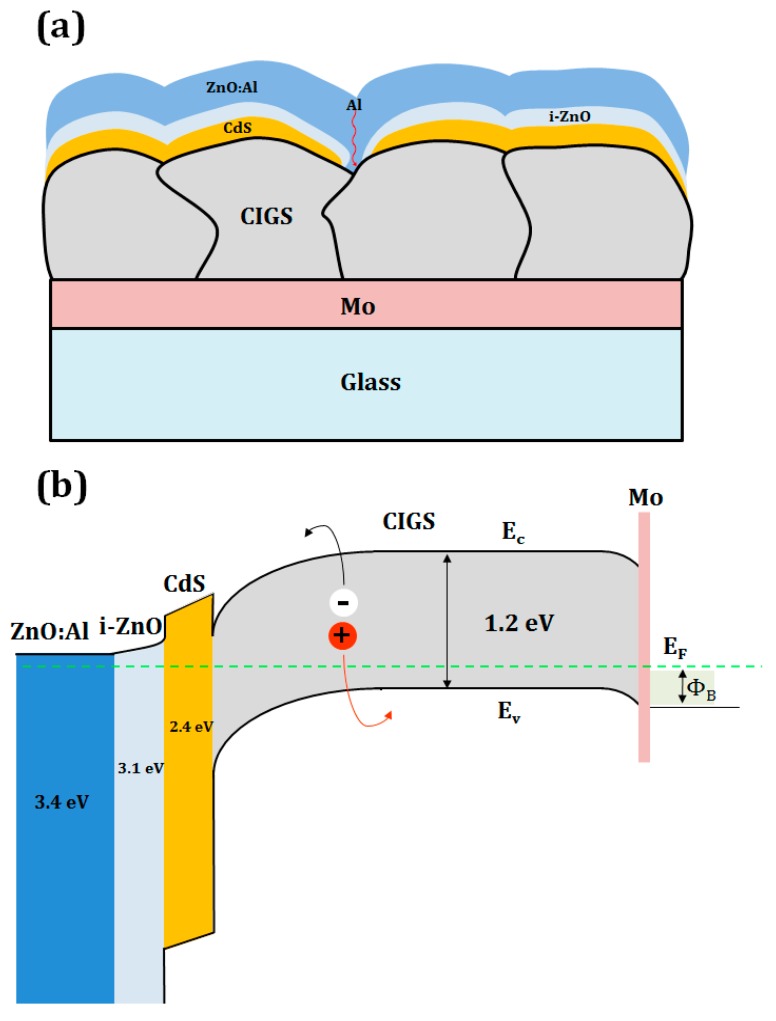
Schematic of (**a**) typical structure and (**b**) energy band diagram of CIGS device. (Modified from Reference [19]).

In the co-evaporation process, the quaternary CIGS films are deposited on the substrate heated to a temperature ranging from 500 to 600 °C by simultaneous or sequential delivery of elemental Cu, In, Ga, and Se fluxes [11]. Conversely, in the two-step metallization-selenization process, the quaternary or quinary Cu(In,Ga)(SeS)_2_ films are produced through the selenization and/or sulfurization reaction of Cu-Ga-In intermetallic precursors, with significant volume expansion and phase evolution [10]. On the deposited glass/Mo/CIGS light absorber layers, a thin cadmium sulfide (CdS) buffer layer, an intrinsic zinc oxide layer (i-ZnO), and an n-type Al-doped zinc oxide layer (AZO) are subsequently added to complete the glass/Mo/CIGS/CdS/i-ZnO/AZO cell structure, as shown in Reference [12]. The typical device structure and energy band diagram of CIGS solar cell are shown in Figure 1. The highly resistive i-ZnO film plays an important role in achieving high-efficiency CIGS solar cells, while working as a shield to protect the CdS/CIGS junction from damage during direct current (DC) sputtering of the highly conductive AZO film [13]. The i-ZnO improves the open-circuit voltage (V_OC_) and fill factor (FF) by reducing the shunt paths [14,15,16]. In general, the shunt path could form by the presence of pinholes in the CdS buffer layer causing the direct contact of conductive elements (e.g., Al, Ga, and B) in transparent conductive oxide (TCO) with CIGS absorber (Figure 1a) and the leakage of current through these shunts’ path [14,17]. Furthermore, an i-ZnO layer forms cliff-like conduction band alignment with a CdS buffer layer and make the generated electron from the CIGS absorber move to front contact effectively [18]. As listed in Table 1, a wide range of i-ZnO thicknesses (50 to 200 nm) has been employed for CIGS solar cells depending on the CIGS deposition processes by different research groups and companies.

In this study, the effects of the i-ZnO thickness on the electrical properties of CIGS devices were fabricated using two different CIGS absorbers with different surface roughnesses evaluated. Furthermore, the correlation between the CIGS device parameters and i-ZnO thickness was investigated.

## 2. Materials and Methods

In this study, two types of CIGS light absorbers prepared by conventional three-stage co-evaporation and sulfurization after selenization (SAS) of a CuGaIn metal precursor were used for subsequent post-processing to fabricate the glass/Mo/CIGS/CdS/i-ZnO/AZO cells. For the deposition of the CdS films with a thickness of approximately 70 nm on the CIGS absorbers, a typical chemical bath deposition (CBD) method was adapted, where cadmium sulfate (CdSO_4_, Sigma-Aldrich, Saint Louis, MO, USA) and thiourea ((H_2_N)_2_CS, Sigma-Aldrich, Saint Louis, MO, USA) were used as the source of the Cd and S ions, respectively. Ammonia hydroxide (NH_4_OH, DUKSAN, Ansan, Gyeonggi-do, Republic of Korea) was added as a complexing agent. A detailed procedure of the CBD used for the CdS deposition is described in a previous study [29].

The deposition of thin i-ZnO layers on glass/Mo/CIGS/CdS samples and bare soda-lime glasses (SLGs) was performed by sputtering a high purity (99.999%), 3″-diameter i-ZnO disc target in a radio frequency (RF) magnetron sputtering system. During the sputtering process, the RF power and base pressure were set to 90 W and 1.6 × 10^−6^ Torr, respectively, while the working pressure was maintained at approximately 8 mTorr with an Ar flow rate of 30 sccm. The substrates were rotated at 60 rpm without heating. The deposition times varied from 10 to 60 min (i.e., 10, 20, 30, and 60 min) at fixed deposition conditions to obtain different thicknesses of the i-ZnO thin films. Lastly, highly conductive AZO (~400 nm thick) and Ni:Ag front grid (~1450 nm thick) were added using DC magnetron sputtering and an electron beam evaporation method, respectively, which creates SLG/Mo/CIGS/CdS/i-ZnO/AZO/Ni:Al solar cells.

Characterization of the SLG/i-ZnO thin films and CIGS devices was achieved using various techniques. X-ray diffraction (XRD: PANalytical X’Pert PRO MPD, Malvern Panalytical, Almelo, Overijssel, Netherlands) with a Cu-K_α1_ radiation of *λ* = 1.54056 Å was used to investigate the crystallographic structure. A Cary 5000 (Varian) double-beam UV-VIS spectrometer (Aglient, Santa Clara, CA, USA) assisted with a non-polarized light at a normal incidence in the wavelength range of 280 to 1280 nm was used for the analysis of the optical transmittance and absorbance at room temperature. Field emission scanning electron microscopy (FE-SEM, Hitachi S-4800, HITACHI, Chiyoda, Tokyo, Japan) was used to characterize the surface morphology and thickness of the i-ZnO thin films. The topography of CIGS films was examined by atomic force microscopy (AFM, Model: XE-100, Park System, Suwon, Gyenggi-do, Republic of Korea). X-ray photoelectron spectroscopy (XPS, ESCALAB 250, Thermo Fisher Scientific, Waltham, MA, USA) was employed to investigate the chemical state of the SLG/i-ZnO samples. The parameters of the illuminated current density and voltage (I-V) characteristics under air mass 1.5 (AM 1.5) one-sun condition were estimated using a K201, LAB 55 Solar Simulator (McScience, Suwon, Republic of Korea) to evaluate the photovoltaic performance of the CIGS devices.

## 3. Results and Discussions

### 3.1. Microstructure and Morphology

**Figure 2:**
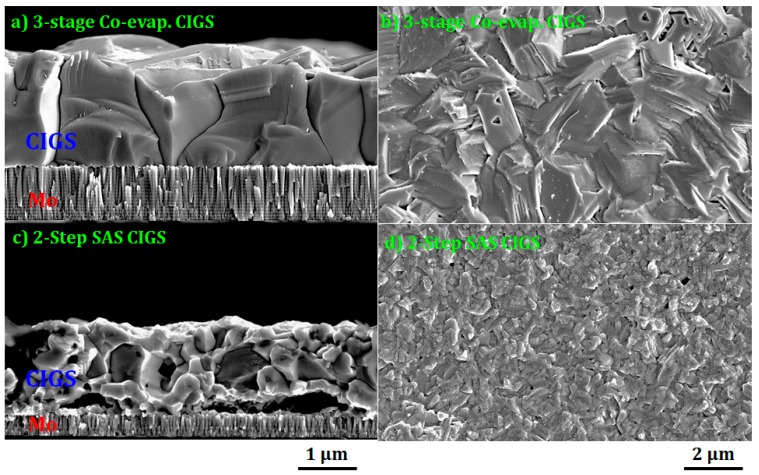
Surface and cross-sectional SEM images of the (**a**,**b**) three-stage co-evaporated CIGS and (**c**,**d**) two-step SAS CIGS.

As shown in Figure 2, the SEM micrographs showed a significant difference in the microstructure and morphology of the CIGS absorbers prepared by three-stage co-evaporation (three-stage co-evaporated CIGS) and two-step sulfurization after selenization of the CuGaIn precursor (two-step SAS CIGS). The three-stage co-evaporated CIGS thin films exhibited a smooth surface with dense and large grains, which is typical for three-stage co-evaporated CIGS [30], while the two-step SAS CIGS films had a rough surface with several voids. As previously reported, the formation of (InGa)_2_Se_3_ at the first stage produced a smooth surface through the three-stage CIGS process [31].

**Figure 3:**
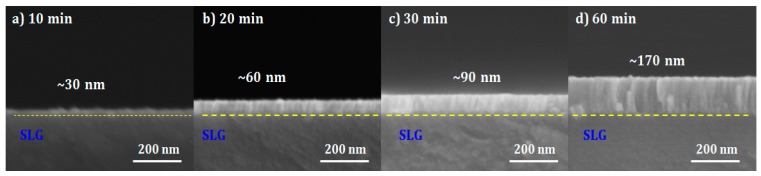
Cross-sectional SEM images of SLG/i-ZnO prepared with different deposition times: (**a**) 10 min, (**b**) 20 min, (**c**) 30 min, and (**d**) 60 min.

**Figure 4:**
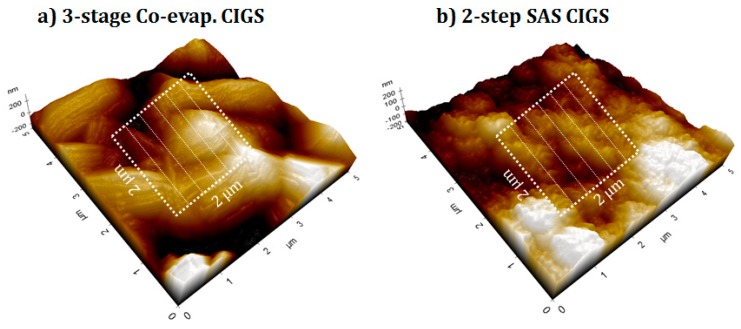
3D AFM images of the (**a**) three-stage co-evaporated CIGS and (**b**) two-step SAS CIGS.

The thicknesses of the i-ZnO films prepared on SLG with different deposition times (i.e., 10–60 min) were estimated by cross-sectional SEM images as shown in Figure 3, where the thickness (30, 60, 90, and 170 nm) of i-ZnO almost linearly increased with the deposition time (10, 20, 30, and 60 min) at a deposition rate of ~0.5 Å/s. With the given resolution of SEM, the 10-min grown i-ZnO film showed a rougher surface morphology than that of the thin thickness at 30 nm. Figure 4 showed the 3D AFM images of the three-stage co-evaporated and the two-step SAS processed CIGS absorbers, where it was demonstrated that the three-stage co-evaporated CIGS showed the smoother surface with bigger grains than the two-step SAS CIGS sample. The root mean square (RMS) surface roughness (Rq) of both CIGS absorbers was calculated for the surface area of 2 × 2 μm^2^ (marked in Figure 4). It was found that the three-stage co-evaporated CIGS had less RMS roughness (Rq ~ 7.8 nm) than the two-step SAS CIGS (Rq ~ 18.7 nm). The results obtained from AFM analysis is in accordance with SEM analysis.

**Figure 5:**
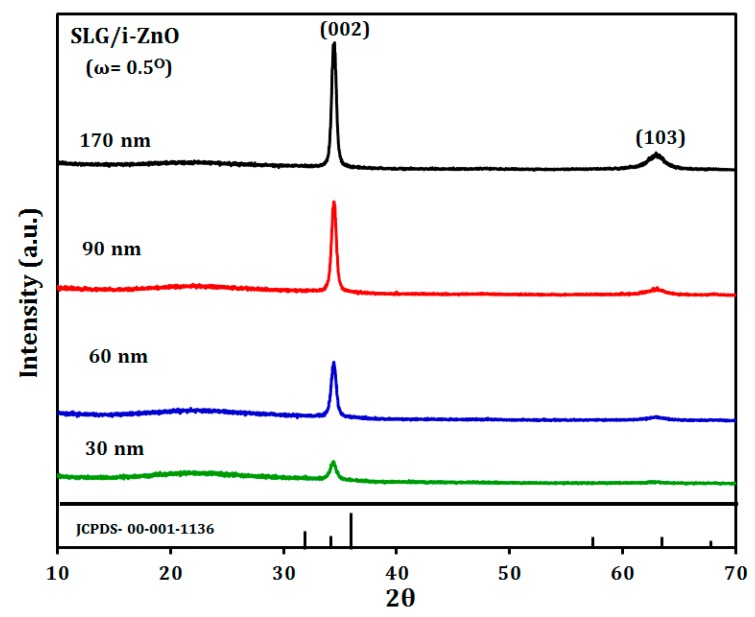
Grazing incidence X-ray diffraction patterns (ω = 0.5°) of the SLG/i- ZnO films prepared with a different thickness.

The crystallographic properties of the SLG/i-ZnO thin films were examined by the grazing incidence XRD (GI–XRD) using Cu-K_α1_ radiation with an incident angle, ω = 0.5°, due to their small thickness in the range of 30 to 170 nm. The diffraction patterns in Figure 5 confirmed that all films had a hexagonal crystal structure with a (002) preferred orientation [32,33], as shown by a relatively high (002)/(103) intensity ratio for our i-ZnO thin films compared to (002)/(103) = 1 for a powder diffraction database of ZnO, Joint Committee on Powder Diffraction Standards (JCPDS) #00-001-1136 card.

**Figure 6:**
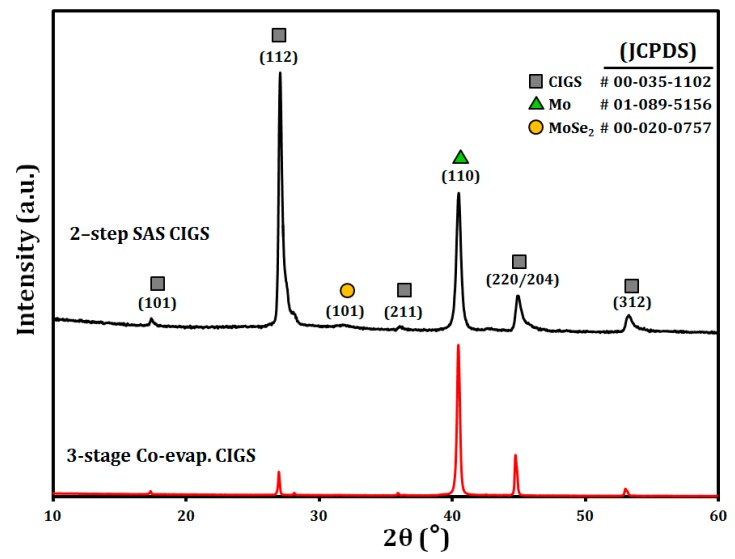
X-ray diffraction patterns of the two-step SAS CIGS (top) and three-stage co-evaporated CIGS films (bottom) grown on the glass/Mo substrates.

Figure 6 shows the bulk XRD reflection patterns of the CIGS absorbers grown on the glass/Mo substrates by three-stage co-evaporation and two-step SAS methods. Both of the samples have a polycrystalline chalcopyrite crystal structure of CIGS with different preferred orientations. As typically reported, the three-stage co-evaporated CIGS film showed a (220) preferred orientation with a (112)/(220) peak intensity ratio of ~0.59, while the two-step SAS CIGS had a (112) preference with a (112)/(220) intensity ratio of ~7.23.

The properties of the CIGS absorber and its cell performance can be influenced by the preferred orientation of the CIGS, i.e., (112) or (220), because the difference in the preferred orientation of the CIGS absorber will cause different effects on the grain boundary activities, which is related to the carrier transport in the CIGS thin-film solar cells [34,35]. The small grains and significant grain boundaries in the CIGS with a (112) preferred orientation represent a strong carrier recombination [36]. Recently, Londhe et al. reported that the CIGS film deposited by electrodeposition with –1.6 V had a (220) preferred orientation with a large grain and high device efficiency (~9.07%), compared to the CIGS electro-deposited at –0.6 V. This shows a (112) preferred orientation with a small grain size and lower efficiency (~4.90%) [37]. As shown in Figure 6, the characteristic X-ray reflection peaks of the two-step SAS CIGS films, e.g., (112), (220/204), and (312), were shifted to a higher 2θ due to the incorporation of the smaller sulfur atoms in the CIGS chalcopyrite crystal structure, which yields reduced lattice constants [38]. Furthermore, MoSe_2_ peaks were observed in the two-step SAS CIGS films. However, these peaks were not visible in the three-stage co-evaporated CIGS, which is a typical characteristic [39].

**Figure 7:**
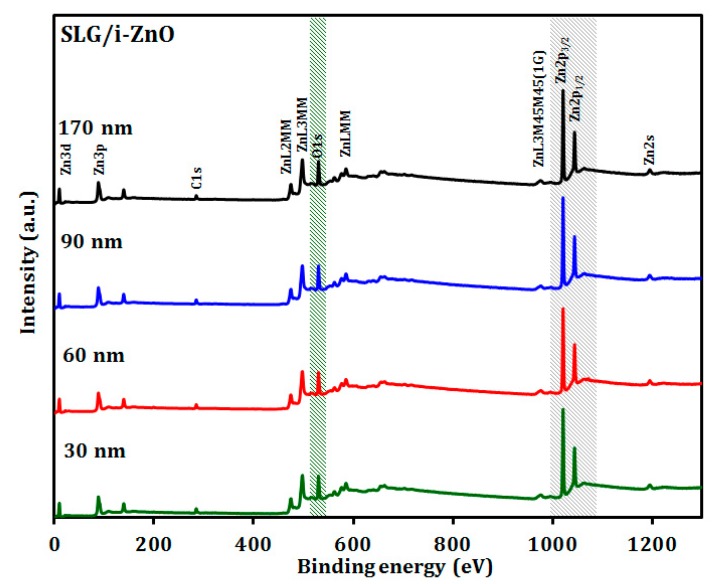
XPS survey spectra of the SLG/i-ZnO thin films with different thicknesses.

**Figure 8:**
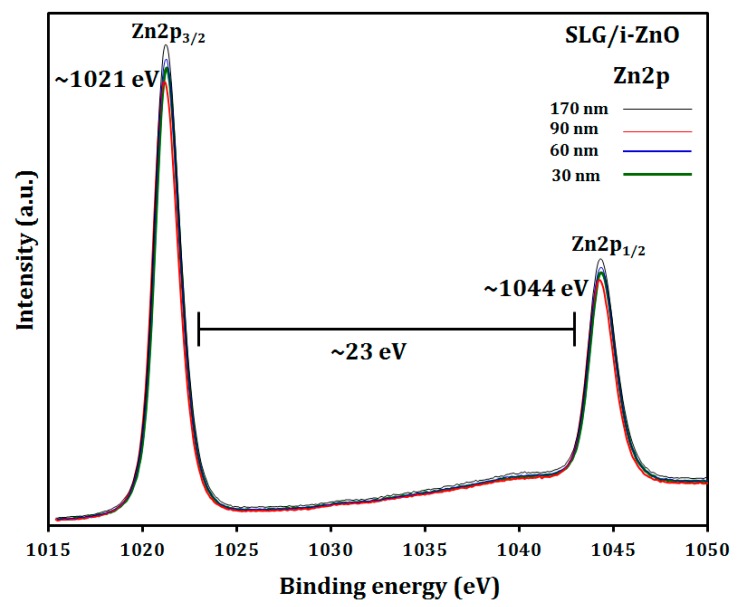
High resolution XPS spectra for the Zn 2p peaks of the SLG/i-ZnO films with different thicknesses in the region highlighted in grey in Figure 7.

**Figure 9:**
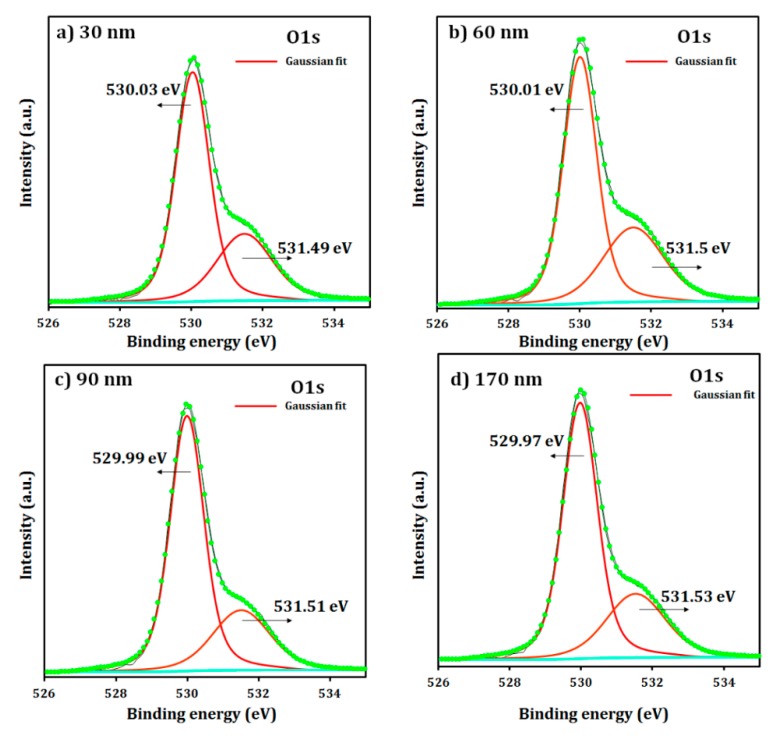
High resolution XPS spectra for the O1s peak of the SLG/i-ZnO films with different thicknesses in the region highlighted in green in Figure 7: (**a**) 30 nm, (**b**) 60 nm, (**c**) 90 nm, and (**d**) 170 nm.

The chemical bonding states of the SLG/i-ZnO films with different thicknesses were investigated by XPS. As shown in Figure 7, the survey spectrum of the SLG/i-ZnO films showed Zn 2p, O 1s, and C 1s energy regions, where a small peak for C 1s was detected at ~284 eV corresponding to the C-C bond, which may arise from the contamination of the sample surface from the surrounding atmosphere [40]. In Figure 8, the high resolution XPS spectra confirmed Zn 2p_1/2_ and Zn 2p_3/2_ peaks, which were located at ~1044 eV and ~1021 eV, respectively, with no significant shift from the film thickness change. A further analysis of the Zn 2p spectra can determine the oxidation state of Zn. The energy difference between the Zn 2p_1/2_ and Zn 2p_3/2_ peaks was ~23 eV, which is in good agreement with the reported value of the Zn^2+^ oxidation state for the ZnO phase [41]. As shown in Figure 9, the asymmetric O 1s peak between 528 eV and 534 eV in the high resolution XPS spectra can be deconvoluted to two Gaussian peaks. The peak at the lower binding energy ~530 eV is attributed to the Zn–O bond, which originated from the O^2−^ ions in the wurtzite lattice structure of ZnO [42,43]. Conversely, the peak at the higher binding energy ~531.5 eV may be attributed to O^2−^ in the oxygen deficient regions of the ZnO matrix [42]. Moreover, the O 1s peak at the higher binding energy 531.5 eV could be attributed to the Zn–OH bond [44]. Some studies have reported that the O 1s peak at the higher binding energy may be attributed to the dissociated or chemisorbed O or OH species on the ZnO thin-film surface, such as the adsorbed H_2_O or O_2_ [45]. There was no noticeable shift for both peaks with the i-ZnO thickness ranging from 30 to 170 nm.

### 3.2. Optical Properties

**Figure 10:**
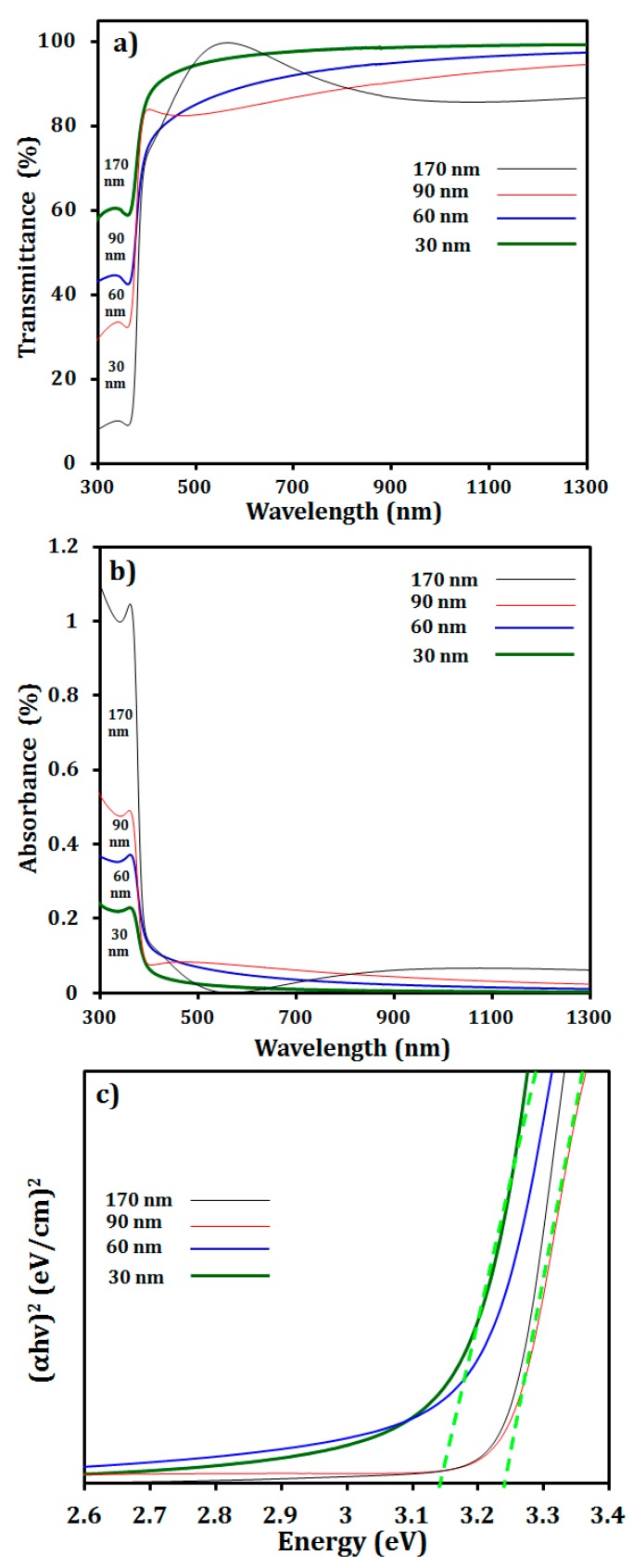
Optical properties of the SLG/i-ZnO thin film measured by UV-VIS spectroscopy: (**a**) transmittance, (**b**) absorbance, and (**c**) optical band gap.

The optical properties of the SLG/i-ZnO films were measured at room temperature at a wavelength ranging from 280 to 1280 nm using a UV-VIS spectrometer. Figure 10a,b shows that the increase in the thickness of the i-ZnO film resulted in a decreased transmittance of the film, whereas the absorbance was increased accordingly. In the short wavelength region, a sudden increase, such as a hill or overshoot near the visible light region, was observed as the thickness of the film increased, e.g., a small hill for 90 nm and larger hill for 170 nm, although a high transparency (˃80%) of all the films was maintained. Similar behaviors showing a hill in the transparency were reported for the film thicker than 100 nm by several researchers [46,47]. The abrupt increase in the transmittance of the thick films in the visible light spectra may be attributed to the interference within the films by the reflection of the lights from the upper and bottom surfaces of the films [47].

Figure 10c shows that the optical band gap of the i-ZnO thin films may slightly shift to a higher band gap energy (e.g., 3.1 to 3.2 eV) with an increasing film thickness from 30 to 170 nm. The slight increase in the optical band gap may be caused by the increase of the carrier concentration with the increased film thickness, which is known as the Burstein–Moss shift [48].

### 3.3. Influence of the i-ZnO Thickness on the CIGS Solar Cell Performance

**Figure 11:**
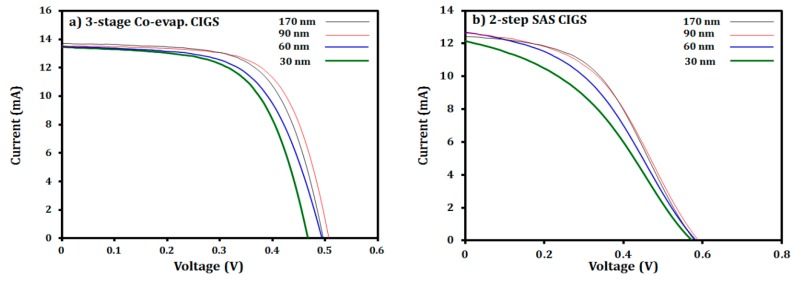
Current and voltage characteristics of the CIGS solar cells with different i-ZnO thicknesses and different CIGS absorbers: (**a**) three-stage co-evaporation CIGS and (**b**) two–step SAS CIGS.

**Figure 12:**
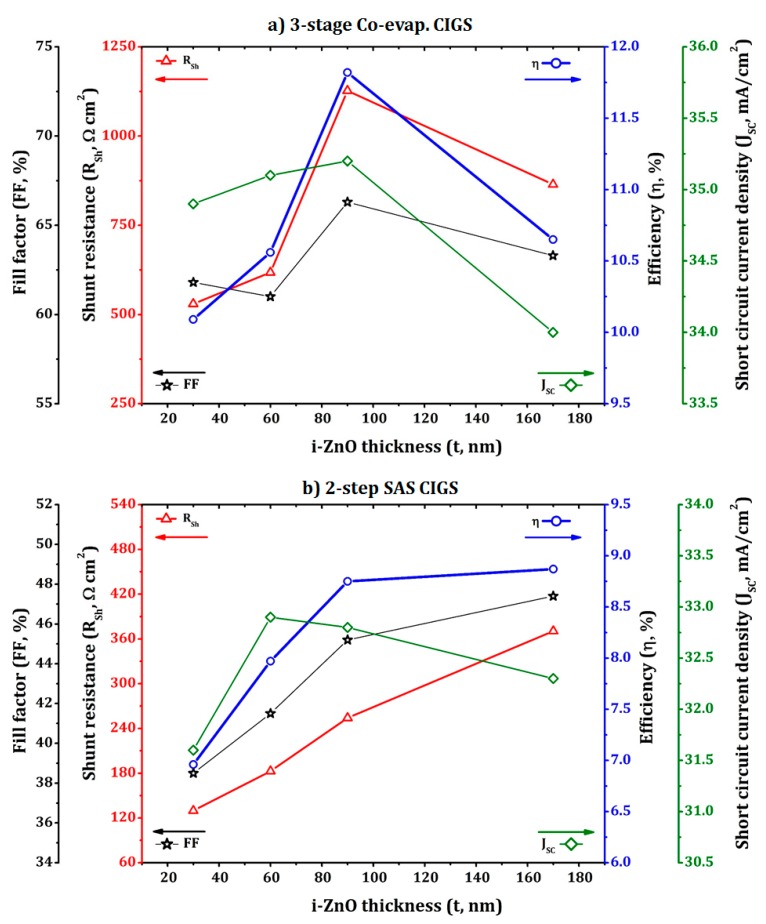
Change of the CIGS device performance parameters, i.e., fill factor (FF), shunt resistance (R_SH_), efficiency (η), and short circuit current density (J_SC_), with the i-ZnO thickness for (**a**) three-stage co-evaporated CIGS and (**b**) two-step SAS CIGS absorbers.

The photovoltaic performance of the glass/Mo/CIGS/CdS/i-ZnO/AZO solar cells fabricated by two different CIGS absorbers, i.e., three-stage co-evaporated and two-step SAS processed CIGS absorbers, as a function of the i-ZnO thickness was compared, as listed in Table 2 and shown in Figure 11 and Figure 12. The results revealed that the change in the i-ZnO thickness affected the performance of the CIGS cells. For the three-stage co-evaporated CIGS cell, as the thickness of the i-ZnO layer increased from 30 nm to 90 nm, the shunt resistance (R_SH_) of the cell was significantly increased from 529.6 to 1126 Ω cm^2^, which results in the improvement of the V_OC_ (from 0.467 V to 0.507 V), FF (from 61.8% to 66.3%) and conversion efficiency (from 10.1% to 11.8%), as displayed in Figure 12a. The improvement in the shunt resistance of the device with the i-ZnO thickness of 90 nm was due to the full coverage of the possible shunt paths within the CIGS/CdS layers with i-ZnO [31,49]. However, a further increas of i-ZnO thickness to 170 nm decreased the device performance parameters, especially the lowest J_SC_ as the thick ZnO layer weakened the built-in field by spreading the space charge region [31]. In addition, the decreased J_SC_ could be attributed to poor transmittance of the 170-nm thick i-ZnO layer and increased series resistance (R_S_) of the resulting device [50].

As shown in Figure 11, the I-V curves for two-step SAS CIGS devices deviated from the normal I-V curve while those for the three-stage co-evaporated CIGS device were close to a normal shape. This deviation is due to the reduced FF with relatively smaller shunt resistance and larger series resistance (see Table 2) [51]. For the device with the two-step SAS processed CIGS absorber, the overall shunt resistance (130–371 Ω cm^2^) was smaller than that of the device with the three-stage co-evaporated CIGS absorber (530–1127 Ω cm^2^), as listed in Table 2. Furthermore, the value of the shunt resistance monotonically increased with the i-ZnO thickness in the range of 30 to 170 nm, as shown in Figure 12b, while the three-stage co-evaporated CIGS cell had a maximum shunt resistance at the 90-nm thick i-ZnO film, as shown in Figure 12a. This trend was caused by the surface of the two-step SAS processed CIGS film that was rougher than the three-stage co-evaporated CIGS films, as confirmed by the SEM results in Figure 2. Therefore, the 90-nm thick i-ZnO layer did not fully cover the potential shunt paths produced during the two-step SAS deposition process of the CIGS absorber. The monotonic increase of the shunt resistance with the i-ZnO thickness resulted in an increase in the conversion efficiency due to the increase of the FF. However, a further increase of the i-ZnO thickness over 170 nm was restricted by the reduced J_SC_ and V_OC_ due to the thick insulating layer of i-ZnO. Therefore, other deposition techniques should be evaluated for obtaining better coverage for the two-step SAS processed CIGS absorber while maintaining a low thickness. The solution processed i-ZnO layer might be more suitable for the rough two-step SAS processed CIGS absorber because the solution process may completely cover the ZnO atoms on the surface by ion-by-ion growth [52].

## 4. Conclusions

The two types of CIGS absorbers prepared by three-stage co-evaporation and two-step SAS methods had different preferred crystal orientations, morphologies, and surface roughnesses. The different surface roughnesses required different optimum thicknesses of the i-ZnO buffer layer to prevent electrical shunt paths in the completed device. The solar cell fabricated by the three-stage co-evaporated CIGS with a smooth surface had an optimum i-ZnO thickness of ~90 nm, while the optimum i-ZnO thickness for optimal solar cell parameters could be proposed to be 170 nm or greater for the cell made with the two-step SAS processed CIGS absorber with a rough surface. However, a further increase of the insulating i-ZnO layer could lead to a decrease of J_SC_ and V_OC_, while positively increasing the shunt resistance.

## Figures and Tables

**Table 1:** Summary of the i-ZnO thickness for different CIGS processes by several research groups and companies.

Organization	Substrate	Absorber Preparation Method	i-ZnO Thickness (nm)	Conversion Efficiency (%)	Note	Reference
ZSW	Glass	Co-evaporation	50–100	22.6		[8]
Solibro GmbH	Glass		50	14.4		[20]
Uppsala	Glass		70	18.6		[21]
EMPA	Glass		80	20.7		[22]
	Steel		50	18	Flexible mild steel	[23]
	polyimide (PI)		50–100	18.7	FlexiblePI	[19]
Nexcis	Glass	Electrodeposition and atmospheric annealing	80	17.3		[24]
IBM	Glass	Solution and spin coating	80	15.2	Hydrazine-based solution	[25]
NREL	Glass	Electrodeposition and selenization (CIGSe)	60	11.7		[26]
	Glass	Evaporation and selenization	50	18.6		[27]
Solar Frontier	Glass	Metal sputtering + Sulfurization after selenization	100–200	18.6	Mini module	[28]

**Table 2:** Performance of the CIGS solar cell fabricated by the three-stage co-evaporated and two-step SAS processed CIGS absorbers as a function of the i-ZnO layer thickness.

Growth Process of CIGS	i-ZnO Thickness (nm)	Solar Cell Performance Parameters					
		J_SC_ (mA/cm^2^)	V_OC_ (V)	FF (%)	Efficiency (%)	R_SH_ (Ω cm^2^)	R_S_ (Ω cm^2^)
Three-stage co-evap.	30	34.9	0.467	61.8	10.1	529.6	7.24
	60	35.1	0.494	61.0	10.6	617.8	8.26
	90	35.2	0.507	66.3	11.8	1127	6.54
	170	34.0	0.495	63.3	10.7	864.5	7.66
Two-step SAS	30	31.6	0.574	38.5	6.96	129.7	31.0
	60	32.9	0.580	41.5	7.97	182.7	28.4
	90	32.8	0.590	45.2	8.75	254.0	25.6
	170	32.3	0.580	47.4	8.87	370.6	24.3
